# An epigenome-wide study of DNA methylation profiles and lung function among American Indians in the Strong Heart Study

**DOI:** 10.1186/s13148-022-01294-8

**Published:** 2022-06-09

**Authors:** Arce Domingo-Relloso, Angela L. Riffo-Campos, Martha Powers, Maria Tellez-Plaza, Karin Haack, Robert H. Brown, Jason G. Umans, M. Daniele Fallin, Shelley A. Cole, Ana Navas-Acien, Tiffany R. Sanchez

**Affiliations:** 1 Integrative Epidemiology Group, Department of Chronic Diseases Epidemiology, National Center for Epidemiology, Carlos III Health Institute, 28029 Madrid, Spain; 2 Department of Environmental Health Sciences, Columbia University Mailman School of Public Health, New York, USA; 3 Department of Statistics and Operations Research, University of Valencia, Valencia, Spain; 4 Millennium Nucleus on Sociomedicine (SocioMed) and Vicerrectoría Académica, Universidad de La Frontera, Temuco, Chile; 5 Department of Computer Science, ETSE, University of Valencia, Valencia, Spain; 6 United States Environmental Protection Agency, Washington, DC USA; 7 Population Health Program, Texas Biomedical Research Institute, San Antonio, TX USA; 8 Department of Environmental Health and Engineering, Johns Hopkins Bloomberg School of Public Health, Baltimore, USA; 9 MedStar Health Research Institute, Hyattsville, MD USA; 10 Georgetown-Howard Universities Center for Clinical and Translational Science, Washington, DC USA; 11 Departments of Mental Health and Epidemiology, Johns Hopkins Bloomberg School of Public Health, Baltimore, USA

**Keywords:** DNA methylation, Lung function, Lung disease, Epigenetics, American Indians

## Abstract

**Background:**

Epigenetic modifications, including DNA methylation (DNAm), are often related to environmental exposures, and are increasingly recognized as key processes in the pathogenesis of chronic lung disease. American Indian communities have a high burden of lung disease compared to the national average. The objective of this study was to investigate the association of DNAm and lung function in the Strong Heart Study (SHS). We conducted a cross-sectional study of American Indian adults, 45–74 years of age who participated in the SHS. DNAm was measured using the Illumina Infinium Human MethylationEPIC platform at baseline (1989–1991). Lung function was measured via spirometry, including forced expiratory volume in 1 s (FEV1) and forced vital capacity (FVC), at visit 2 (1993–1995). Airflow limitation was defined as FEV1 < 70% predicted and FEV1/FVC < 0.7, restriction was defined as FEV1/FVC > 0.7 and FVC < 80% predicted, and normal spirometry was defined as FEV1/FVC > 0.7, FEV1 > 70% predicted, FVC > 80% predicted. We used elastic-net models to select relevant CpGs for lung function and spirometry-defined lung disease. We also conducted bioinformatic analyses to evaluate the biological plausibility of the findings.

**Results:**

Among 1677 participants, 21.2% had spirometry-defined airflow limitation and 13.6% had spirometry-defined restrictive pattern lung function. Elastic-net models selected 1118 Differentially Methylated Positions (DMPs) as predictors of airflow limitation and 1385 for restrictive pattern lung function. A total of 12 DMPs overlapped between airflow limitation and restrictive pattern. *EGFR*, *MAPK1* and *PRPF8* genes were the most connected nodes in the protein–protein interaction network. Many of the DMPs targeted genes with biological roles related to lung function such as protein kinases.

**Conclusion:**

We found multiple differentially methylated CpG sites associated with chronic lung disease. These signals could contribute to better understand molecular mechanisms involved in lung disease, as assessed systemically, as well as to identify patterns that could be useful for diagnostic purposes. Further experimental and longitudinal studies are needed to assess whether DNA methylation has a causal role in lung disease.

**Supplementary Information:**

The online version contains supplementary material available at 10.1186/s13148-022-01294-8.

## Introduction

Between 1980 and 2014, the mortality rate for chronic respiratory disease, including chronic obstructive pulmonary disease (COPD) and interstitial lung disease (ILD), increased by 29.7% in the U.S [1]. COPD is defined by airflow limitation that is not fully reversible [2], whereas ILD is defined by the presence of cellular proliferation, infiltration and/or fibrosis of the lung not due to infection or neoplasia and resembles a restrictive spirometry pattern [3]. The development of chronic lung disease is associated with both environmental and genetic risk factors. Although cigarette smoking is one of the main risk factors for chronic lung disease development, not every smoker will develop chronic lung disease and many patients with chronic lung disease have never smoked.

Epigenetic modifications, including DNA methylation (DNAm), are often related to environmental exposures, and are increasingly recognized as key processes in the pathogenesis of chronic lung disease [4–8]. In a systematic review examining the association of lung function with global, epigenome-wide, and locus-specific DNAm in peripheral blood from population-based studies, five of the six included studies showed evidence that DNAm profiles were differentially associated with lung function, including loci associated with the *SERPINA1*, *ORC4*, *WT1*, and *FXYD1* genes [9]. *SERPINA1,* for example, encodes alpha-1-antitrypsin, and alpha-1-antitrypsin deficiency has been shown to cause degenerative pulmonary disease through unregulated tissue breakdown [10]. Evidence suggests that DNAm alterations could play a role in the predisposition to or pathogenetic mechanism of lung disease. While there is a growing number of studies that evaluate the association of lung disease and differential DNAm profiles, epidemiologic studies examining lung disease-related DNAm profiles of American Indian communities are scarce.

The objective of this study was to investigate the association of DNAm with lung function and spirometry-defined lung disease in the Strong Heart Study (SHS). We used elastic-net models to select relevant CpGs, and conducted a bioinformatic analysis to evaluate the biological plausibility of the findings.

## Methods

### Study population

**Fig. 1:**
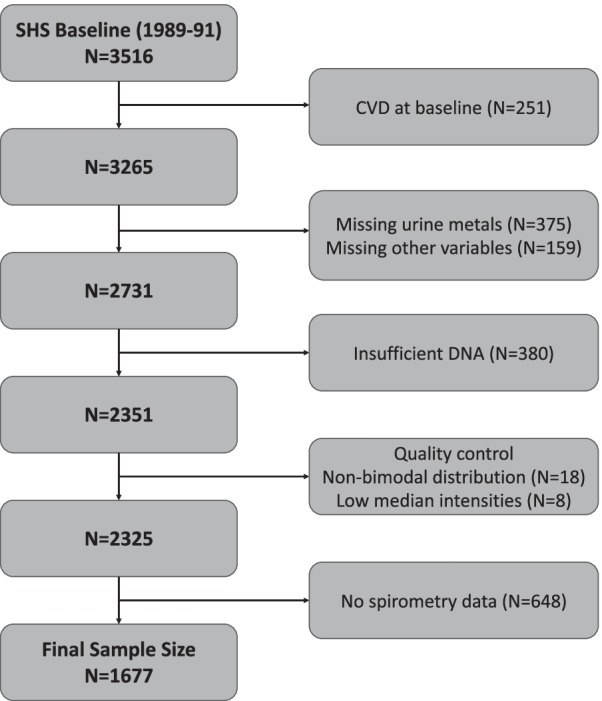
Flowchart of included participants. *5 participants missing education data, 2 smoking status, 11 BMI, 52 LDL cholesterol, 14 hypertension treatment, 111 eGFR, 30 diabetes

The SHS is a prospective cohort study funded by the National Heart, Lung and Blood Institute and the National Institute of Environmental Health Sciences to investigate cardiovascular disease and its risk factors in American Indian adults [11]. In 1989–1991, 4549 men and women aged 45–75 years, members of 13 tribes based in Arizona, Oklahoma, and North Dakota and South Dakota who were free of cardiovascular disease enrolled in the study. DNAm was measured in 2351 participants at the SHS baseline visit (1989–1991). Details regarding inclusion criteria for blood DNAm measurements have been described elsewhere [12]. Among eligible participants with DNAm data, participants without a valid spirometry test at visit 2 (1993–1995) were excluded (*N* = 648), as were individuals missing relevant covariate information, leaving a total of 1677 participants in this study (Fig. 1).

### Participant characteristics

At baseline, trained and certified nurses and medical examiners administered a standardized questionnaire and physical examination including collecting information on sociodemographic (age, sex, study region, education level), lifestyle (smoking status), medical history (prior tuberculosis infection) and anthropometric (height and weight) factors. A fasting blood sample was also collected during the physical examination.

### Spirometry and self-reported lung disease

Pre-bronchodilator spirometry testing was conducted by centrally trained and certified nurses and technicians. Maneuvers were considered acceptable according to then-current American Thoracic Society recommendations [13]. Spirometry reference values for SHS participants have been previously derived [14]. Spirometry endpoints include absolute measures of forced expiratory volume in 1 s (FEV1) and forced vital capacity (FVC), FEV1/FVC, and fixed ratio-defined airflow limitation (FEV1/FVC < 0.70) and restriction (FVC < 80% predicted, FEV1/FVC > 0.70). For fixed-ratio defined lung disease endpoints, participants with FEV1/FVC > 0.7 and FVC > 80% predicted served as the reference group.

### Blood DNA methylation determinations

Details of microarray DNAm measurements at the baseline visit of the SHS have been published elsewhere [12]. Briefly, DNAm from white blood cells was measured using the Illumina MethylationEPIC BeadChip (850 K). CpGs with a *p*-detection value greater than 0.01 in more than 5% of the individuals (6159 CpGs) were removed. In addition, cross-hybridizing probes, probes located in sex chromosomes and Single Nucleotide Polymorphisms (SNPs) with minor allele frequency > 0.05 were excluded. Single sample snoob normalization and regression on correlated probes normalization were conducted following Illumina’s recommendations for preprocessing (*minfi* and *Enmix* R packages) [15]. Blood cell proportions (CD8T, CD4T, NK cells, B cells, monocytes and neutrophils) were estimated using the *FlowSorted.Blood.EPIC* R package [16]. Beta values, which range from 0 to 1 and represent the proportion of unconverted cytosines (Cs) in bisulfite-converted DNA at specific locations, were calculated using the R package *minfi*. [15] We used all cell types except neutrophils (the most common cell type) as adjustment variables in regression models. We detected and corrected for potential batch effects by sample plate, sample row, and DNA isolation time with the combat function (*sva* R package) [17]. We conducted annotation of CpGs to the nearest gene according to the Infinium MethylationEPIC Manifest File v1.0b4 [18, 19]. CpG sites that were not annotated to any gene according to Illumina’s manifest files were annotated to the closest gene using the matchGenes function from the bumphunter R package. The preprocessing resulted in data from 1677 individuals and 788,368 CpG sites in our analyses.

### Statistical analysis

#### Differentially methylated positions (DMPs) analysis by elastic net

We examined five outcomes: (1) FEV1 (in Liters) as a continuous variable, (2) FVC (in Liters) as a continuous variable, (3) FEV1/FVC (%) as a continuous variable, (4) airflow limitation versus normal lung function as a dichotomous variable, and (5) restrictive versus normal lung function as a dichotomous variable. Given that many smoking-related genes were found to be DMPs for airflow limitation, we repeated the analysis among never smokers, both as self-reported and as identified by the EpiSmokEr tool [20], which predicts smoking status using DNAm data. In contrast to traditional one-by-one linear regression CpG modeling approaches, which are limited in accounting for large numbers of predictors or correlated data, we used elastic-net. Elastic-net methods have recently become very popular in Epigenome-Wide and Genome-Wide Association Studies [21–23] as the elastic-net method is robust to limitations of the Lasso method such as dealing with multicollinearity in very high-dimensional settings [24, 25]. Specifically, when the correlations among predictors are high, the elastic-net method exceeds the predictive accuracy of the Lasso [26]. Elastic-net has previously shown to be able to select relevant predictors in differential DNAm analysis and has been used to construct methylation-based risk-scores that have shown great promise for disease prediction based in epigenetic data. [21, 27, 28]

We used elastic-net to select DMPs (simultaneously modeled independent variables) that were associated with lung function and spirometry-defined lung disease (dependent variables). Among the DMPs selected by elastic-net, we then ran traditional linear regression models (for continuous outcomes) and logistic regression models (for dichotomous outcomes) for each CpG separately to obtain effect estimates and 95% CI-s.

Elastic-net, linear and logistic models were adjusted for smoking status (never, former, current), cumulative smoking (cigarette pack-years), age, squared age, sex, BMI, study center (Arizona, Oklahoma or North Dakota and South Dakota), prior tuberculosis diagnosis [29] and cell counts (CD8T, CD4T, NK, B cells and monocytes). For continuous, absolute measures of lung function, we also adjusted for height. To account for population stratification, models were additionally adjusted for five genetic principal components (PCs) [30]. Of 2562 genotyped SHS participants as part of the CALiCo/PAGE Study, we identified 644 unrelated individuals (either founders of pedigrees or unrelated spouses of their descendants). Of 162,718 autosomal SNPs that passed quality control, we selected 15,158 based on the following criteria: minor allele frequency ≥ 0.05, minimum physical separation of 1 kb, and pairwise correlation of genotype scores ≤ 0.1 within a 100 kb sliding window. We performed PC analysis on the genotype scores within unrelated individuals using the R function prcomp. The first five PCs were kept as adjustment variables, as they explained most of the variance. Multiple comparisons were accounted separately using the Benjamini and Hochberg method for false discovery rates (FDR).

To assess whether results were affected by family relatedness, we ran a sensitivity analysis and repeated the linear and logistic models for each of the five lung function measures restricted to unrelated individuals (i.e., selecting only one individual within each family). In this sensitivity analysis, we additionally excluded individuals with mismatches in reported sex vs sex predicted using DNA methylation data as computed by the getSex function from the minfi R package. [15]

#### Protein–protein interaction network

From the DMPs selected in the elastic-net models, we created two sets of protein-coding genes. The first set represents the airflow limitation phenotype using the following 3 outcomes: FEV1, FEV1/FVC, and airflow limitation vs. normal lung function. The second set represents a restrictive phenotype using the following 3 outcomes: FEV1, FVC, and restrictive vs. normal lung function. The protein interaction information was obtained from the STRING database v11.0 [31]. The STRING database provides a confidence score (from 0 to 1) obtained from the estimated likelihood of each annotated interaction between a given pair of proteins being biologically meaningful, specific and reproducible [31]. The protein interaction networks were analyzed and displayed using the yfiles Organic layout by Cytoscape v. 3.7.2 [32]. In the resultant networks, we only kept connections obtained from experimental studies with a minimum confidence score of 0.4. The unconnected nodes were excluded from the network. We also conducted PPI network enrichment analysis in the resultant networks.

#### Enrichment analyses

We used the EWAS Toolkit [33] to test for trait enrichment for each of the five lung endpoints. The CpGs selected by the elastic-net models were introduced in the EWAS Toolkit separately for airflow limitation, restrictive pattern, FEV1, FVC and FEV1/FVC. In addition, we used the ToppCluster tool [34] for comparative enrichment among gene clusters selected by elastic-net for the five endpoints. CpG sites were annotated to the closest gene using the matchGenes function from the R package bumphunter, and then were introduced in the Toppcluster tool in five clusters (one per endpoint). Enriched Gene Ontology terms within and between clusters were checked at a Bonferroni-corrected p-value of 0.05. In addition, we introduced the top genes selected by elastic-net as well as the most connected nodes in the protein–protein interaction network into the GWAS Catalog [35] to test whether they had been identified in previous genome-wide association studies.

## Results

**Fig. 2:**
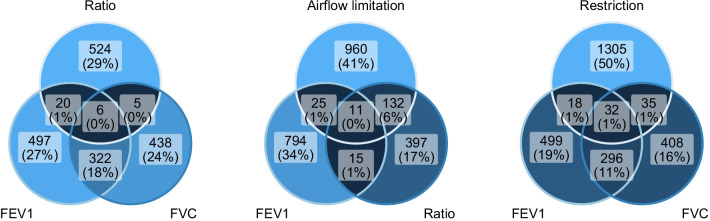
Venn diagram of elastic-net selected DMPs for lung function outcomes, airflow limitation, and restrictive pattern

1677 participants were included (Fig. 1). Participant’s characteristics are presented in Table 1. At baseline (time of blood collection), all participants were 44–75 years of age (mean 55 years old). 61% of participants were female, and 32% had never smoked. The elastic-net model selected 838 DMPs for FEV1, 762 DMPs for FVC, 545 DMPs for FEV1/FVC, 1118 DMPs for airflow limitation and 1385 for restrictive pattern lung function. 328 of the DMPs selected for FEV1 (38.8%) overlapped with selected DMPs for FVC elastic-net models, whereas 26 DMPs (3.1%) overlapped with FEV1/FVC (Fig. 2). Airflow limitation shared 36 DMPs with FEV1 (3.2%), 143 DMPs with FEV1/FVC (12.7%) and 11 DMPs overlapped together with FEV1, FEV1/FVC and airflow limitation (Fig. 2). Restrictive pattern lung function shared 50 DMPs with FEV1 (2.9%), 67 DMPs with FVC (4.8%) and 32 DMPs overlapped between FEV1, FVC and restrictive pattern lung function (Fig. 2), while 12 DMPs overlapped between airflow limitation and restrictive pattern, no DMPs overlapped with all 5 outcomes.

**Table 1:** Participant characteristics

	Overall (*N* = 1677)	Airflow limitation (*N* = 352)	Restriction (*N* = 229)	Non-cases (*N* = 1096)
Female, %	61	51	70	62
Age (years), median (IQR)	55 (49, 61)	59 (52, 65)	56 (49, 61)	53 (48, 60)
Height (cm), median (IQR)	165 (159, 173)	168 (160, 176)	163 (158, 169)	165 (160, 173)
BMI, median (IQR)	29.7 (26.3, 33.8)	27.7 (24.3, 31.5)	31.5 (27.3, 35.7)	29.9 (26.8, 33.8)
Smoking, *n* (%)				
Never	32	26	37	32
Former	30	26	28	32
Current	38	48	35	36
Center, %				
Arizona	14	7	27	14
North Dakota and South Dakota	43	29	47	46
Oklahoma	43	63	27	40
eGFR, median (IQR)	100.7 (92.1, 107.5)	97.7 (88.3, 105.2)	101.7 (93.4, 107.9)	101.1 (93.1, 108.0)
Education (years), median (IQR)	12 (10, 14)	11 (9, 13)	12 (9, 13)	12 (10, 14)
Prior TB diagnosis, %	13	18	15	12
FEV1 (L), median (IQR)	2.5 (2.0, 3.0)	2.1 (1.6, 2.7)	1.9 (1.6, 2.2)	2.7 (2.3, 3.2)
FVC (L), median (IQR)	3.3 (2.7, 4.0)	3.4 (2.7, 4.4)	2.3 (1.9, 2.8)	3.4 (2.9, 4.1)
FEV1/FVC, median (IQR)	76.5 (71.1, 75.3)	65.2 (59.2, 68.2)	80.3 (75.7, 85.9)	77.8 (74.8, 81.4)

*IQR* Interquartile range, *eGFR* Estimated glomerular filtration rate, *TB* Tuberculosis, *FEV1* Forced expiratory volume in 1 s, *FVC* Forced vital capacity

Table 2 shows the top five DMPs selected by the elastic-net models and the mean differences (95% CIs) for continuous lung function measures (FEV1, FVC, FEV1/FVC) comparing percentile 90 to percentile 10 of DNA methylation calculated using linear regression models. Table 3 shows the top five DMPs selected by the elastic-net models and the Odds Ratios (95% CIs) for airflow limitation and restrictive pattern comparing percentile 90 to percentile 10 of DNA methylation calculated using logistic regression models. A list of all DMPs selected by elastic-net models for each of the five lung function outcomes studied are included in Additional file 1: Tables S1–S5. Additional file 2: Fig. S1 shows the distribution of DNA methylation proportions by lung disease status of the top five DMPs for restrictive pattern and the top five DMPs for airflow limitation.

**Table 2:** Top five differentially methylated positions for continuous lung function measures (FEV1, FVC, and FEV1/FVC)

CpG	Chr	Gene	Function	Mean difference (95% CI)	*p* value
*FEV1*					
cg25325512	6	*PIM1*	Serine/Threonine-Protein Kinase Pim-1. Cell proliferation and survival	0.22 (0.15, 0.29)	2.91E−09
cg26058502	1	*CERS2*	Regulation of cell growth and lipid metabolism	0.18 (0.11, 0.25)	2.79E−07
cg01641754	5	*LOC100289230*	Uncharacterized	0.18 (0.11, 0.24)	3.46E−07
cg15167811	9	*PTBP3*	Regulator of cell differentiation	0.16 (0.1, 0.23)	4.85E−07
cg07941411	3	*CD80*	T-cell proliferation and cytokine production	− 0.15 (− 0.21, − 0.09)	2.00E−06
*FVC*					
cg02203833	7	*CDK5*	Serine/threonine kinase. Synaptic plasticity and neuronal migration	0.21 (0.12, 0.30)	4.00E−06
cg19265480	1	*NBPF8*	Associated with developmental and neurogenetic diseases	− 0.19 (− 0.27, − 0.11)	3.00E−06
cg18140268	1	*NBPF8*	Associated with developmental and neurogenetic diseases	− 0.18 (− 0.26, − 0.11)	5.00E−06
cg07343418	12	*GRIN2B*	Brain development, synaptic plasticity	− 0.19 (− 0.26, − 0.11)	2.00E−06
cg03725414	16	*CRISPLD2*	Promotes matrix assembly	0.18 (0.10, 0.26)	1.30E−05
*FEV1/FVC*					
cg25001882	14	*NRXN3*	Cell adhesion in the nervous system, synaptic plasticity	33.0 (21.3, 44.6)	3.32E−08
cg16771344	9	*NTRK2*	Tyrosine receptor kinase. Phosphorylates itself and members of the MAPK signaling pathway	29.9 (18.5, 41.2)	2.99E−07
cg12420787	2	*CACNB4*	Calcium channel. Plasticity on the brain	21.7 (13.4, 30.1)	3.84E−07
cg03636183	19	*F2RL3*	Blood coagulation, inflammation and response to pain. Associated with smoking and lung cancer	18.1 (11.0, 25.2)	5.80E−07
cg27127773	16	*SLX4*	Repair of specific types of DNA lesions	− 42.6 (− 59.2, − 25.9)	6.17E−07

Top five DMPs selected by elastic-net models. Mean differences reported from linear models comparing percentile 90 vs percentile 10 of DNA methylation. Models were adjusted for age, squared age, height, prior tuberculosis diagnosis, sex, BMI, smoking status (never, former, current), cumulative smoking (pack-years), study center (Arizona, Oklahoma, or North and South Dakota), cell counts (CD8T, CD4T, NK, B cells and monocytes) and five genetic PCs

**Table 3:** Top five differentially methylated positions for airflow limitation and restrictive pattern lung function

CpG	Chr	Gene	Function	Odds Ratio (95% CI)	*p* value
*Airflow limitation*					
cg20304857	2	*GALNT14*	Catalyze transfer of N-acetyl-D-galactosamin to hydroxyl groups on serines and threonines	2.8 (1.9, 4.3)	E−06
cg07842459	1	*CD84*	Homophilic adhesion molecule	2.8 (1.8, 4.3)	2E−06
cg17916980	16	*CPPED1*	Serine/Threonine-Protein Phosphatase. Dephosphorylates AKT family kinase	2.5 (1.7, 3.7)	2E−06
cg03647068	2	*FMNL2*	Morphogenesis, cytokinesis, cell polarity	3.0 (1.9, 4.8)	2E−06
cg06100532	16	*LMF1*	Involved in the maturation of specific proteins in the endoplasmic reticulum	2.8 (1.8, 4.3)	3E−06
*Restrictive pattern*					
cg05504535	10	*ADARB2*	Regulatory role in RNA editing	0.3 (0.2, 0.5)	1.86E−07
cg04890495	11	*MCAM*	Cell adhesion, cohesion of the endothelial monolayer at intercellular junctions in vascular tissue	0.3 (0.2, 0.5)	5.92E−07
cg03320255	19	*ZNF540*	Transcriptional repressor	0.3 (0.2, 0.5)	8.72E−07
cg20024687	8	*TOP1MT*	Role in the modification of DNA topology	0.4 (0.2, 0.5)	3.00E−06
cg19693031	1	*TXNIP*	Protects cells from oxidative stress	0.4 (0.3, 0.6)	4.00E−06

Top five DMPs selected by elastic-net models. Odds Ratios reported from regression models comparing percentile 90 vs percentile 10 of DNA methylation. Models were adjusted for age, squared age, prior tuberculosis diagnosis, sex, BMI, smoking status (never, former, current), cumulative smoking (pack-years), study center (Arizona, Oklahoma, or North and South Dakota), cell counts (CD8T, CD4T, NK, B cells and monocytes) and five genetic PCs

Of the 1677 individuals included in this study, 1142 were from unique families (i.e., unrelated). When comparing self-reported sex vs sex predicted using DNA methylation, seven additional individuals presented sex mismatch and were excluded in sensitivity analysis, leaving 1135 participants. Among those, 155 had restrictive pattern disease and 262 had airflow limitation. The OR-s and mean differences when excluding sex-mismatched and related participants were very similar to those of the main analysis (Additional file 1: Tables S1–S5).

**Fig. 3:**
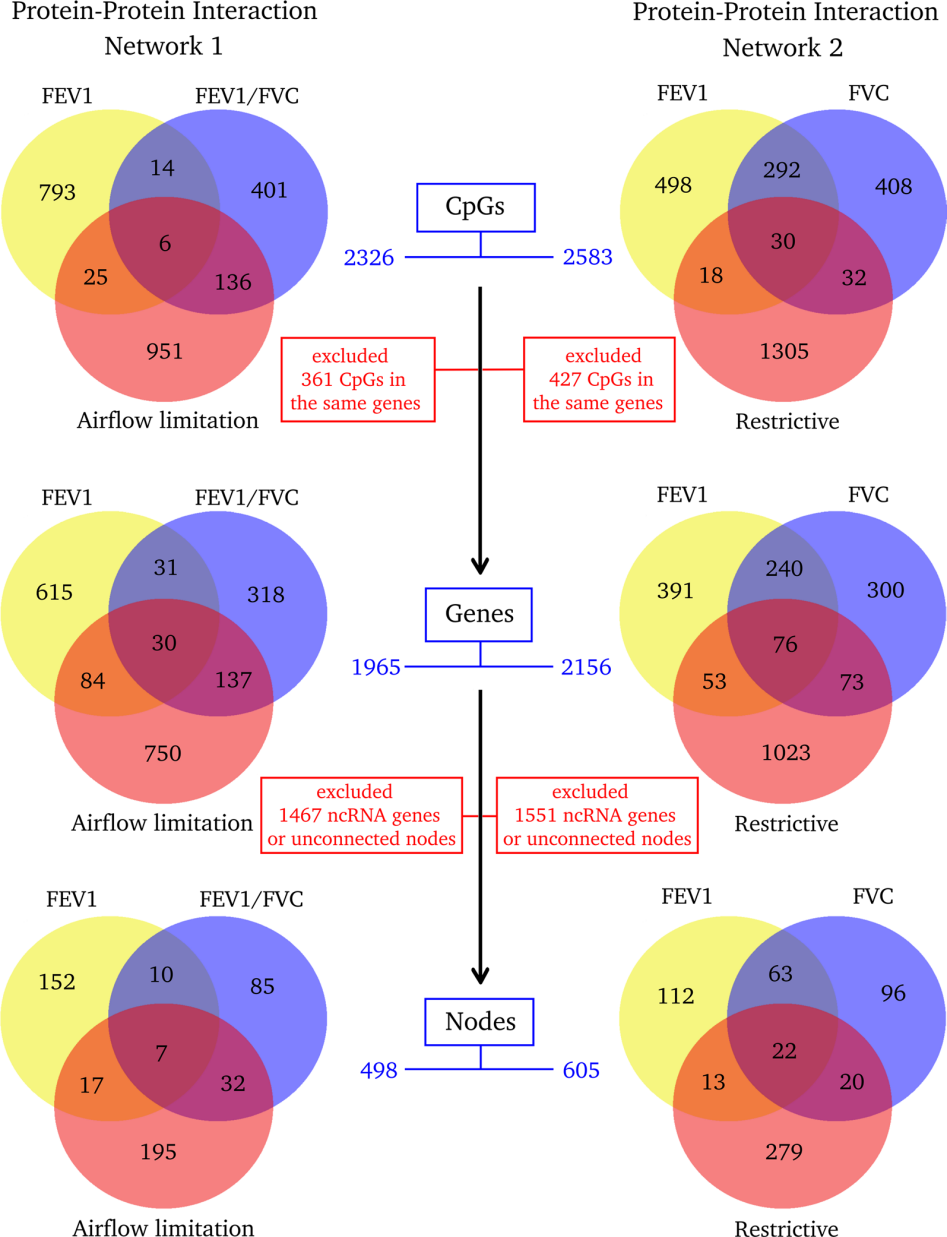
Workflow for protein–protein interaction networks

In the protein–protein interaction networks, the obstructive phenotype network (FEV1, FEV1/FVC and airflow limitation vs normal lung function) included 1965 unique genes associated with 2326 DMPs identified by elastic-net models. Of these, 1467 non-coding RNA genes or unconnected nodes were discarded (Fig. 3, network 1). The protein–protein interaction network for airflow limitation included 498 nodes and 829 interactions (Additional file 3: Fig. S2 and Additional file 1: Tables S6, S7). *EGFR*, *MAPK1* and *PRPF8* were the most connected nodes in the network with 32, 22 and 19 interactions, respectively. The restrictive phenotype network (FEV1, FVC, and restrictive pattern vs normal lung function) included 2156 unique genes associated with 2583 DMPs identified by elastic-net models. Of these, 1551 ncRNA genes or unconnected nodes were discarded (Fig. 3, network 2). The protein–protein interaction network for restrictive pattern included 605 nodes and 1101 interactions (Additional file 4: Fig. S3 and Additional file 1: Tables S8, S9). *UBA52*, *CREBBP*, *SRC* and *EGFR* were the most connected nodes with 38, 34, 29 and 27 interactions, respectively. The PPI network enrichment analysis identified a total of 204 and 355 Gene Ontology (GO) terms significantly enriched (FDR < 0.05) for airflow limitation (network from Additional file 3: Fig. S2, Additional file 1: Table S10) and for restrictive pattern (network from Additional file 4: Fig. S3, Additional file 1: Table S11), respectively.

**Fig. 4:**
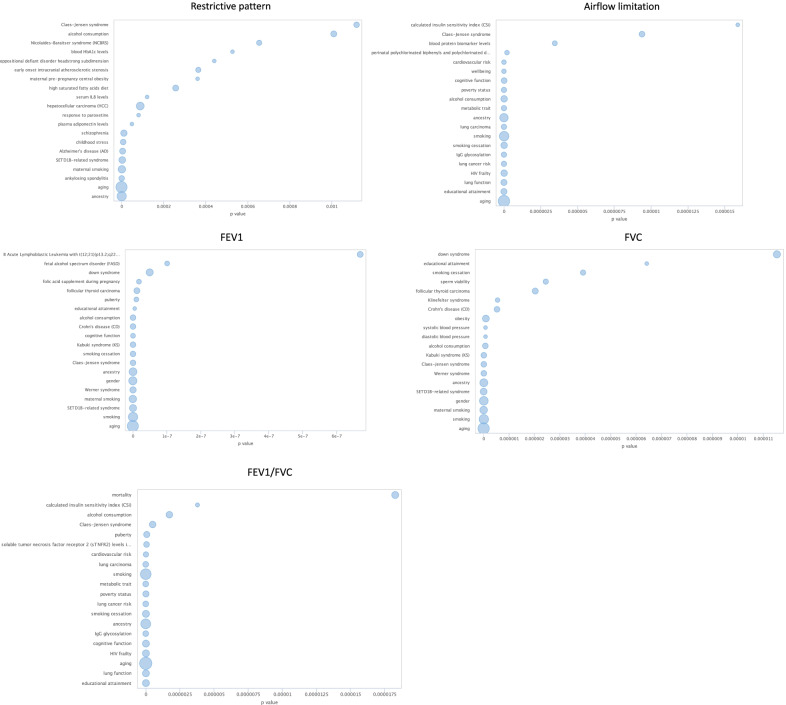
Top enriched traits for FEV1, FVC, FEV1/FVC, airflow limitation and restrictive pattern from the EWAS Toolkit

Figure 4 shows the top enriched traits for each of the five endpoints. Several lung-related traits were enriched: lung function for FEV1/FVC and airflow limitation; smoking, smoking cessation or maternal smoking for all five endpoints, and lung carcinoma for FEV1/FVC and airflow limitation. Additional file 1: Table S12 shows the results from the Toppcluster algorithm. No Gene Ontology terms were commonly enriched across all five endpoints. The Gene Ontology term “animal organ morphogenesis” was enriched for all endpoints except for FEV1/FVC. In addition, the terms “head development", “neuron projection development", “brain development” and “synaptic signaling” were enriched for three endpoints. Other 95 Gene Ontology terms were enriched at Bonferroni 0.05 significance level for two or one lung endpoints. Among the top genes annotated to DMPs identified in our elastic-net models, only the gene EGFR was present in the GWAS Catalog, associated with lung adenocarcinoma.

Given that several smoking-related genes (*AHRR, F2RL3, PRSS23, RARA*) were found to be DMPs for airflow limitation, we repeated that analysis restricted to self-reported never smokers and restricted to those that were classified as never-smokers by the EpiSmokEr tool. There were *N* = 531 self-reported never smokers in our study, of which 92 presented airflow limitation. Eight hundred and sixty-nine CpGs were selected by elastic-net as DMPs. The elastic-net model selected two CpGs annotated to *AHRR*; however, it did not select any CpG annotated to *F2RL3*, *PRSS23* or *RARA* (Additional file 1: Table S13). There were *N* = 848 participants classified by EpiSmokEr as never-smokers. Of those, 139 presented airflow limitation. 756 CpGs were selected by elastic-net. No CpGs annotated to any of the smoking-related genes were selected (Additional file 1: Table S14). The number of overlapping CpGs between the two never-smoker models was 90. The number of overlapping CpGs between the overall population model and the model restricted to never-smokers as classified by EpiSmokEr was 136.

For restrictive pattern, the model was only run for never-smokers as classified by the EpiSmokEr tool, due to lack of power for running it in self-reported never-smokers. There were 121 restrictive pattern cases. 1070 CpGs were selected by elastic-net. The number of overlapping CpGs between the overall population model and the model restricted to never-smokers was 253 (data not shown). Two of the top CpG sites in the overall population model (annotated to genes *ADARB2* and *ZNF540*) were also selected for the model restricted to never-smokers.

Table 4 shows the effect estimates and p-values of the CpGs identified in a meta-analysis [36] that were replicated in the SHS (annotated to genes *AHRR, F2RL3, ALPPL2, IER3, GPR15, SOCS3, TMEM184B* and *CDKN1B*).

**Table 4:** Effect estimates and *p* values of the CpGs identified in the meta-analysis by Imboden et al. that were replicated in the SHS

CpG	Chr	Gene	Lung function endpoint in meta-analysis	Coefficient (SE) in meta-analysis	*p* value in meta-analysis	Lung function endpoint in the SHS	Coefficient of the elastic-net model in the SHS
cg05575921	5	*AHRR*	FEV1; FVC; FEV1/FVC	0.78 (0.057); 0.37 (0.064); 0.12 (0.008)	1.48E−43; 8.79E−09; 7.22E−50	FEV1/FVC	1.10
cg03636183	19	*F2RL3*	FEV1; FVC; FEV1/FVC	1.27 (0.098); 0.62 (0.11); 0.20 (0.015)	4.08E−39; 2.04E−08; 4.5E−43	FEV1/FVC	1.13
cg21566642	2	*ALPPL2*	FEV1; FEV1/FVC	0.94 (0.074); 0.15 (0.011)	7.43E−37; 5.02E−43	FEV1/FVC	0.21
cg03329539	2	*ALPPL2*	FEV1; FEV1/FVC	1.61 (0.15); 0.26 (0.023)	1.46E−26; 5.58E−30	FEV1/FVC	0.76
cg24859433	6	*IER3*	FEV1/FVC	0.30 (0.034)	2.05E−19	FEV1	0.019
cg19859270	3	*GPR15*	FEV1; FEV1/FVC	2.74 (0.36); 0.47 (0.06)	1.34E−14; 2.8E−18	FEV1/FVC	2.46
cg18181703	17	*SOCS3*	FEV1	1.19 (0.16)	3.74E−14	FEV1; FVC	0.093; 0.034
cg01127300	22	*TMEM184B*	FEV1	0.85 (0.12)	1.19E−12	FEV1/FVC	0.057
cg06826457	12	*CDKN1B*	FEV1	1.12 (0.18)	5.35E−10	Airflow limitation; FEV1/FVC	− 0.028; 0.13

## Discussion

We conducted an epigenome-wide association study investigating the association between DNA methylation and lung function and explored common epigenetic signatures between lung function and disease. Using robust methods for high-dimensional correlated data, we found 1118 DMPs associated with airflow limitation and 1385 associated with restrictive pattern lung function. A total of 12 DMPs overlapped between airflow limitation and restrictive pattern. The biological functions of the top genes, as well as the most connected nodes in the protein–protein interaction network, were related to biological processes associated with lung disease.

Several top genes and highly connected nodes in the protein–protein interaction networks for FEV1 (*PIM1*), FVC (*CDK5*), FEV1/FVC (*NTRK2*), airflow limitation (*CPPED1*) and restrictive pattern (*EGFR, MAPK1*) are protein kinases. In addition, the GO term “Positive regulation of transmembrane receptor protein serine/threonine kinase signaling pathway” (GO:0090100) was found to be significantly enriched (FDR = 0.0119) for the restrictive lung function phenotype. Protein kinases play a role in many key pulmonary cellular responses, including mediating inflammatory signals and airway remodeling. Thus, they have been proposed as therapeutic targets for several lung diseases such as chronic obstructive pulmonary disease and asthma [37, 38]. *PIM1* and *CKD5*, top genes associated with FEV1 and FVC, respectively, are serine/threonine protein kinases. An animal study reported evidence of high-tidal volume ventilation increasing pulmonary fibrosis in acute lung injury via the serine/threonine protein kinase B [39]. Also, the mitogen-activated protein kinase 1 gene (*MAPK1*) was a highly connected node in the airflow limitation protein–protein interaction network. Lung endothelial barrier function is regulated by multiple signaling pathways, including mitogen-activated protein kinases (MAPK) [40]. MAPK kinases might contribute to ameliorate the lung endothelial barrier-disruptive effects. [41]

The *EGFR* gene, which was among the most connected nodes in the protein–protein interaction networks for both airflow limitation and restrictive pattern, is a protein kinase. It plays an essential role in pulmonary physiology by regulating key cellular processes such as self-renew, wound-healing, proliferation, survival adhesion, migration and differentiation [42]. *EGFR* inhibitors have been widely used in treatment of non-small cell lung cancer, in fact, it was the first biomarker identified as a potential therapeutic target for personalized treatment in lung cancer. *EGFR* was also identified as associated with lung adenocarcinoma in the GWAS Catalog [35]. DNA methylation in *EGFR* has been proposed as a predictive biomarker for lung adenocarcinoma. Our results suggest that DNA methylation levels in *EGFR* might be predictive of airflow limitation and restrictive pattern as well. Prospective studies of DNA methylation changes and lung disease are needed to assess the potential predictive ability of *EGFR* in lung disease.

On the other hand, the *CRISPLD2* gene, which was a DMP associated with both FEV1 and FVC in our study, was identified in a whole genome sequencing study in children with asthma as associated with FEV1/FVC [43]. Many experimental and human studies have highlighted the importance of the *CRISPLD2* gene in fetal lung regulation, branching morphogenesis and alveologenesis, among other lung function related biological processes [44–46]. Other genes identified in our study are also associated with biological processes relevant for lung function. The Ubiquitin A-52 Residue Ribosomal Protein Fusion Product 1 gene (*UBA52*), for instance, was a highly connected node in the restrictive pattern protein–protein interaction network. Ubiquitination regulates the proteins that modulate the alveolocapillary barrier and the inflammatory response, therefore playing an important role in acute lung injury [47]. Also, the Adenosine Deaminase RNA Specific B2 gene (*ADARB2*) was the top differentially methylated position for restrictive pattern. An animal model showed that adenosine deaminase deficiency might lead to pulmonary fibrosis [48]. Our work provides further evidence that these biological processes are involved in lung disease and can be measured systemically. However, experimental studies are needed to disentangle whether DNAm changes influence these biological pathways or, conversely, alterations in these pathways lead to DNAm dysregulations.

Of note, there was very little overlap between the DMPs associated with airflow limitation and with restrictive pattern (only 12 CpGs), and there was no overlap between the five lung function measures. This fact as well as the fact that the top DMPs associated with restrictive pattern and with airflow limitation (Table 3) had opposite directions of association with DNA methylation, might point to different biological pathways being involved in airflow limitation and restrictive pattern. Importantly, hypomethylation of several smoking-related genes was associated with airflow limitation in our study (*AHRR, F2RL3, PRSS23, RARA*), whereas none of those was associated with restriction. Previous literature have pointed out that hypomethylation in the gene *AHRR*, the most well known smoking-related gene, might be associated with lower lung function and respiratory symptoms [49, 50]. Also, DNAm dysregulation in *AHRR* was associated with lung function in two multi-cohort epigenome-wide association studies in adults [36, 51]. When running the airflow limitation analysis only among self-reported never smokers, elastic-net selected CpGs annotated to the *AHRR* gene, but not to the other three smoking-related genes. However, when running the airflow limitation analysis among never-smokers as classified by the EpiSmokEr tool, no smoking-related genes were selected. Second-hand smoke exposure might be responsible for the effect of *AHRR* in airflow limitation among self-reported never smokers. Further studies are needed to investigate the role of the *AHRR* gene in lung disease beyond smoking.

This is, to our knowledge, the first epigenome-wide association study with the main focus on lung function conducted in a population of American Indians. We found four previous epigenome-wide association studies of lung function with spirometry measurements in other adult populations. One did not report any significant associations (*N* = 1091) [52]. The second one was conducted in a population of female twins in 2012, and only found one DMR associated with FEV1 and FVC annotated to the gene *WT1*, which was not replicated in our population [53]. In 2018, another EWAS of lung function was conducted in two cohorts. Three CpG sites associated with lung function were consistently found in the two cohorts [51]. Only one (cg05575921, annotated to *AHRR*) was replicated in our study. Last, another EWAS was conducted in eight cohorts (three discovery cohorts and five replication cohorts) in 2019 [36], our results are highly consistent with the findings of this recent EWAS, with nine CpGs associated with lung function being replicated in our population (Table 4), and many more at the gene level. Although several other epigenome-wide association studies in lung function have been conducted, they were conducted in specific populations such as children [54], individuals with chronic obstructive pulmonary disease [55], individuals with HIV [56] or never smokers [57]. Findings for these specific populations might not be generalizable. Nevertheless, many differentially methylated positions found in these studies overlapped with our findings. For instance, eight of the top sites found in the never-smokers EWAS of lung function were replicated in our population, which might indicate that the epigenomic signature of lung function is also stable across different population groups. A meta-analysis was conducted among Mexican American and Puerto Rican children [58]. Among the genes identified, only the gene *TBC1D16* was replicated in our population as associated with restrictive pattern. In addition, another meta-analysis conducted by Machin et al. did not find any consistent CpG sites across the six articles assessed for either chronic obstructive pulmonary disease or lung function, which suggests that part of the epigenomic signature of lung function might also be specific to populations. [9]

This work has some limitations. First, only 1677 of the SHS participants were included, which might induce some bias among those who were excluded due to not meeting spirometry quality standards. We were also unable to use the lower limit of normal to classify airflow limitation and restriction due to sample size contraints. In addition, we only have one measure of spirometry and we lack other clinical information, therefore, we cannot discard potential measurement errors. DNA methylation is highly cell-type specific and results from blood cells might not be comparable to DNA methylation in other tissues such as lung. However, the biological plausibility of the findings suggests that blood DNA methylation might be relevant for chronic lung disease. Longitudinal and experimental studies are needed to assess the directionality of the findings. Strengths of this work include using one of the largest methylation arrays currently available in microarray technology (the EPIC array), the large sample size in an indigenous population, measurement of spirometry-defined lung disease, and innovative statistical methods that allow evaluating the effect of methylation sites jointly instead of individually.

In conclusion, we found several differentially methylated positions for FEV1, FVC, FEV1/FVC, obstructive pattern and restrictive pattern, with several genes pointing to biological pathways related to lung disease including protein kinases, which are therapeutic targets for lung disease. Further studies are needed to investigate the potential mechanistic role of DNAm in lung disease.

## Supplementary Information

**Additional file 1. Table S1**. DMPs selected by elastic-net for FEV1. **Table S2.** DMPs selected by elastic-net for FVC.** Table S3**. DMPs selected by elastic-net for FEV1/FVC.** Table S4**. DMPs selected by elastic-net for airflow limitation.** Table S5**. DMPs selected by elastic-net for restrictive pattern.** Table S6.** Protein-protein interaction network nodes for airflow limitation.** Table S7.** Protein-protein interaction network edges for airflow limitation.** Table S8.** Protein-protein interaction network nodes for restrictive pattern.** Table S9.** Protein-protein interaction network edges for restrictive pattern.** Table S10.** Protein-protein interaction network enrichment analysis for airflow limitation. **Table S11.** Protein-protein interaction network enrichment analysis for restrictive pattern. **Table S12.** Toppcluster enrichment for FEV1, FVC, FEV1/FVC, airflow limitation and restrictive pattern.** Table S13.** DMPs selected by elastic-net for airflow limitation restricted to self-reported never smokers.** Table S14.** DMPs selected by elastic-net for airflow limitation restricted to never smokers as reported by EpiSmokEr.

**Additional file 2. Figure S1.** Distribution of DNA methylation proportions by lung disease status of the top five differentially methylated positions for restrictive pattern and the top five DMPs for airflow limitation.

**Additional file 3. Figure S2.** Protein-protein interaction network for airflow limitation phenotype: FEV1, FEV1/FVC and airflow limitation vs normal lung function.

**Additional file 4. Figure S3.** Protein-protein interaction networks for restrictive lung function phenotype: FEV1, FVC and restrictive vs normal lung function.

## Data Availability

The data underlying this article cannot be shared publicly in an unrestricted manner due to limitations in the consent forms and in the agreements between the Strong Heart Study tribal communities and the Strong Heart Study investigators. The data can be shared to external investigators following the procedures established by the Strong Heart Study, available at https://strongheartstudy.org/. All analyses were conducted in R version 3.6.2, and all packages used are freely available in the CRAN repository.
