# Supplementary figures and images for: An epigenome-wide study of DNA methylation profiles and lung function among American Indians in the Strong Heart Study
Source: Clin Epigenetics. 2022 Jun 9;14:75. doi: 10.1186/s13148-022-01294-8 (PMC9185990; doi:10.1186/s13148-022-01294-8)

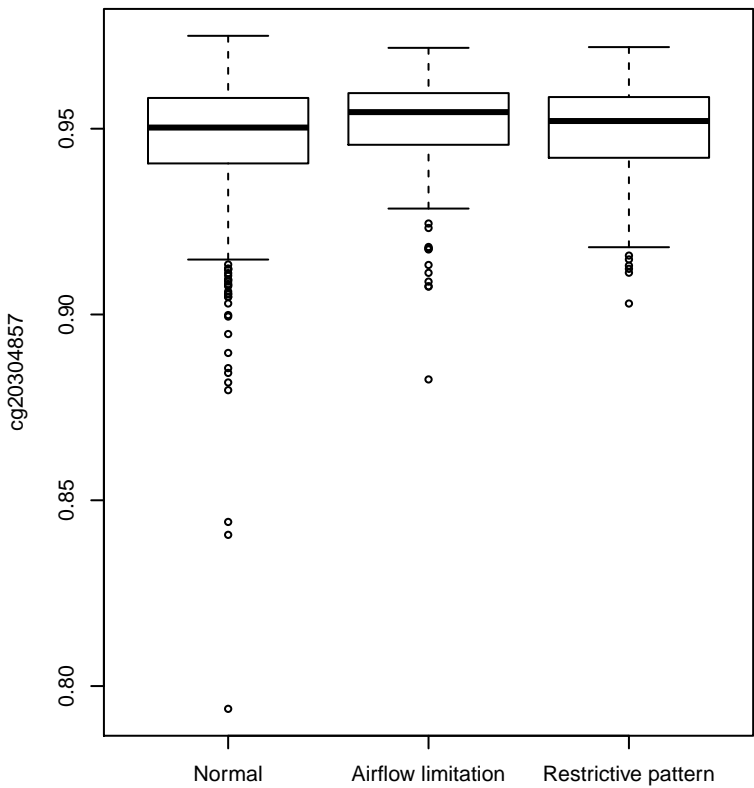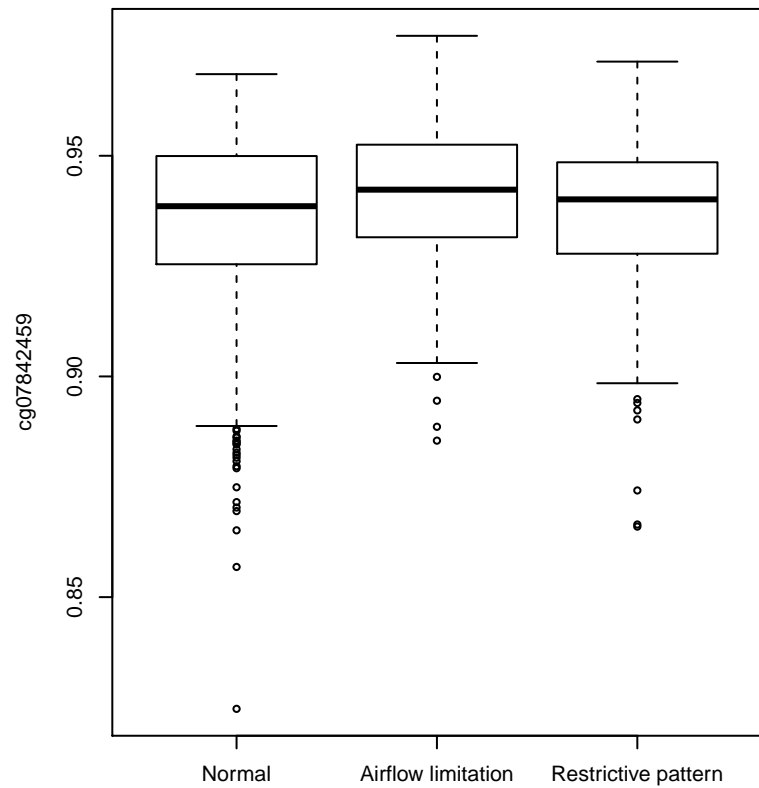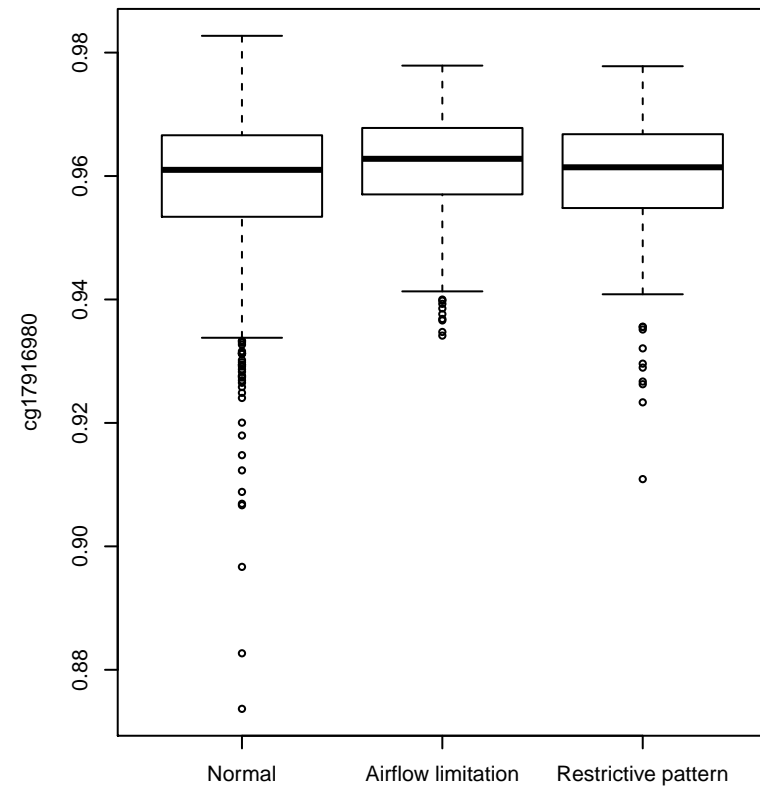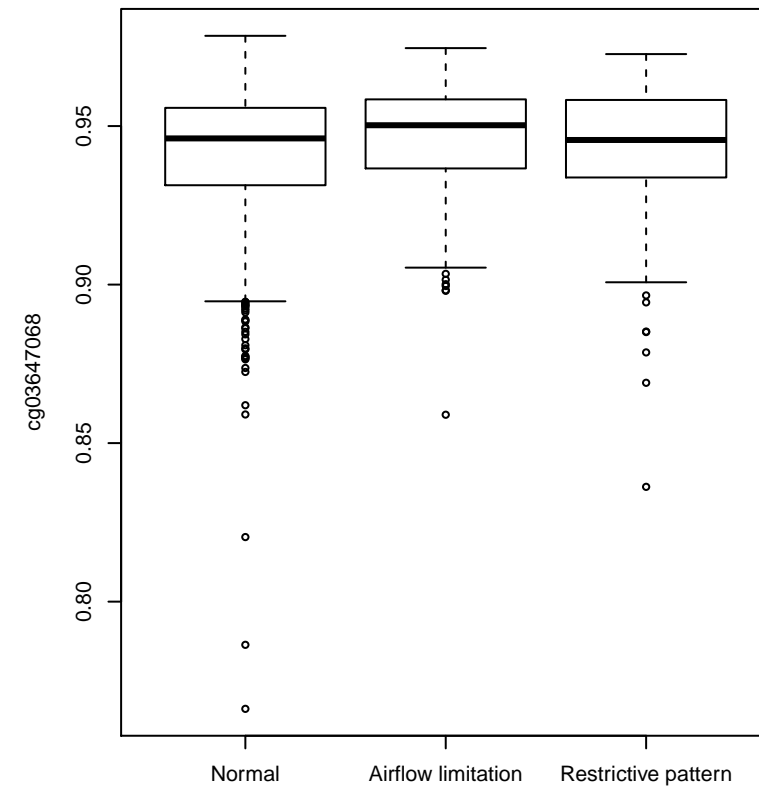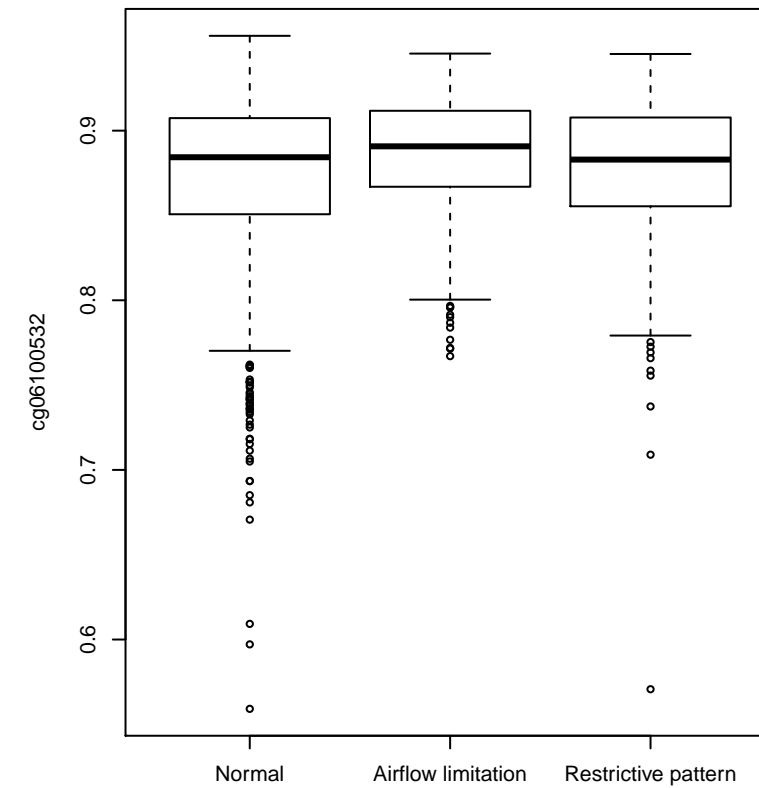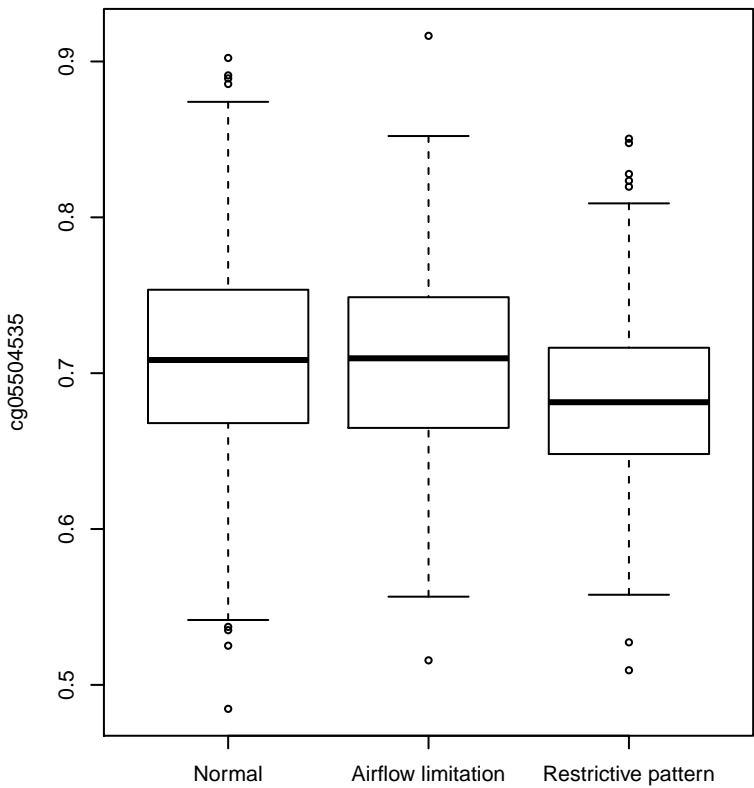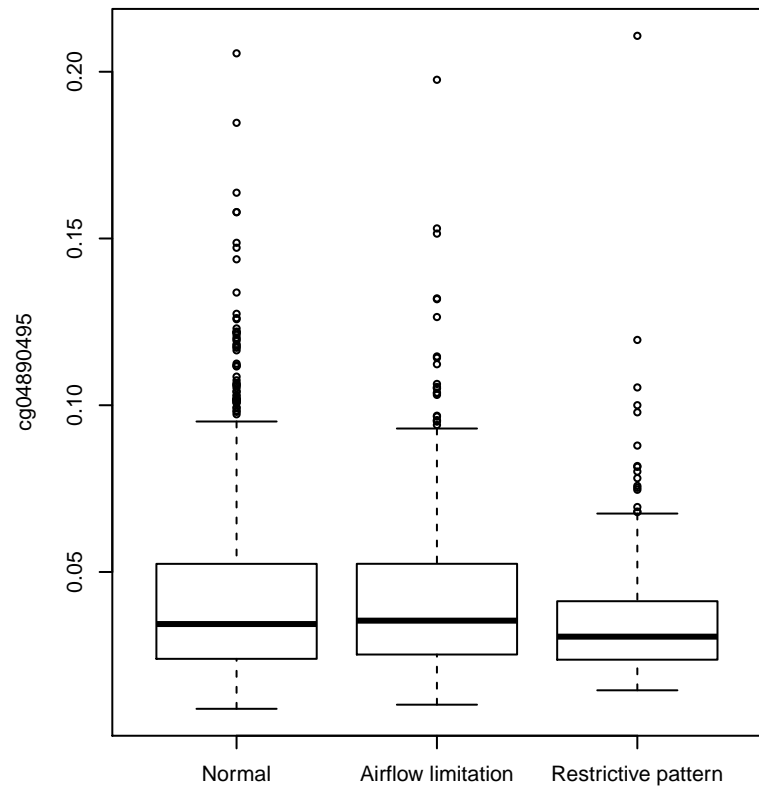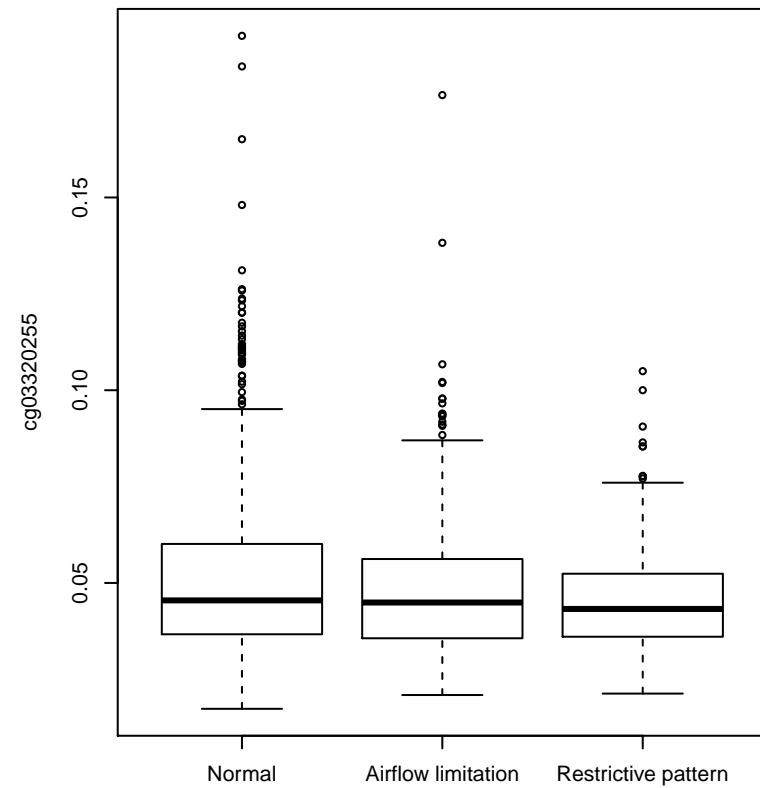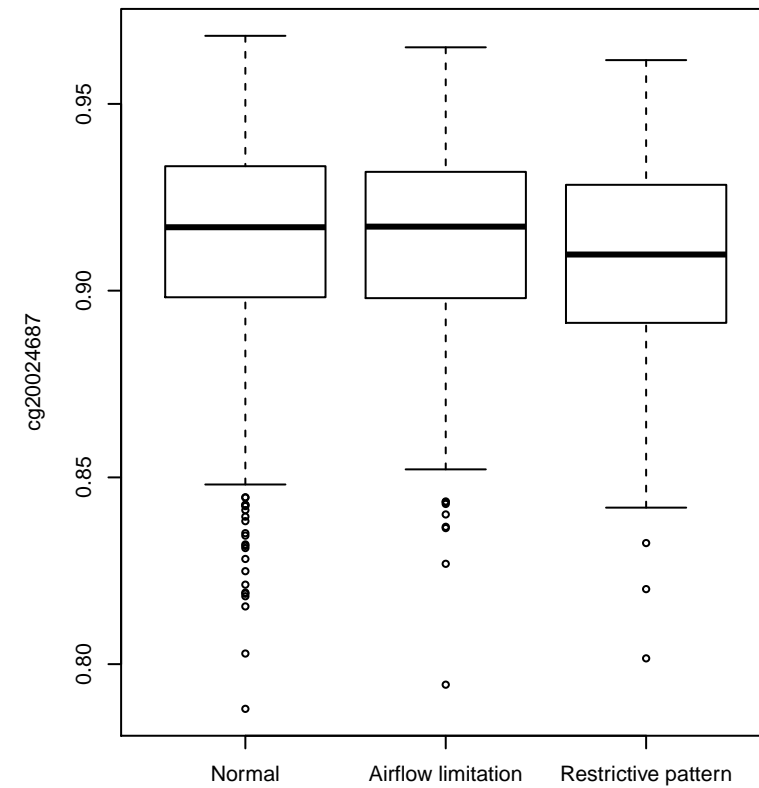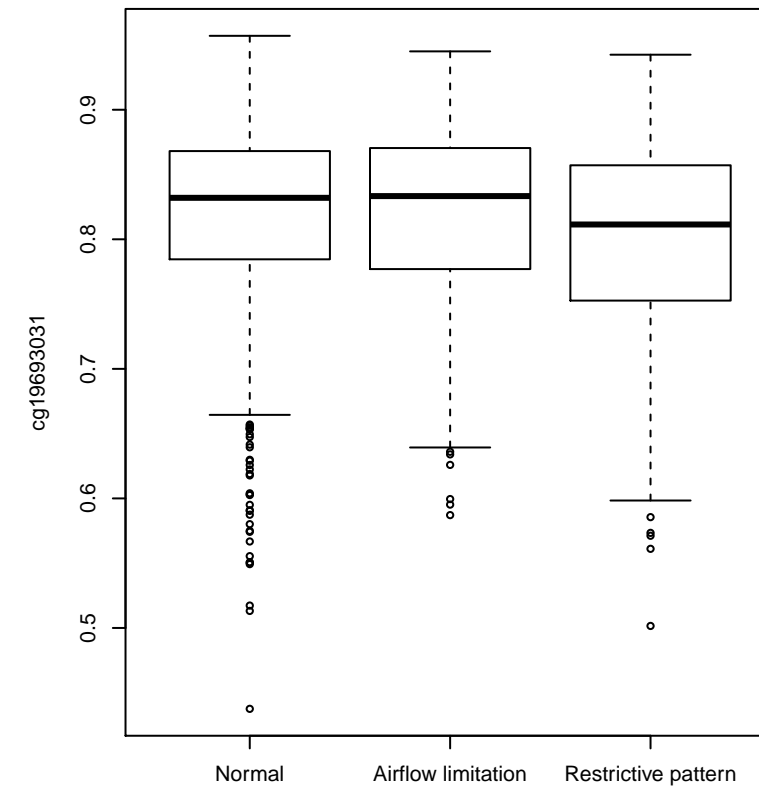

Supplement: Supplementary file 2 — Additional file 2. Figure S1. Distribution of DNA methylation proportions by lung disease status of the top five differentially methylated positions for restrictive pattern and the top five DMPs for airflow limitation. [file 13148_2022_1294_MOESM2_ESM.pdf]

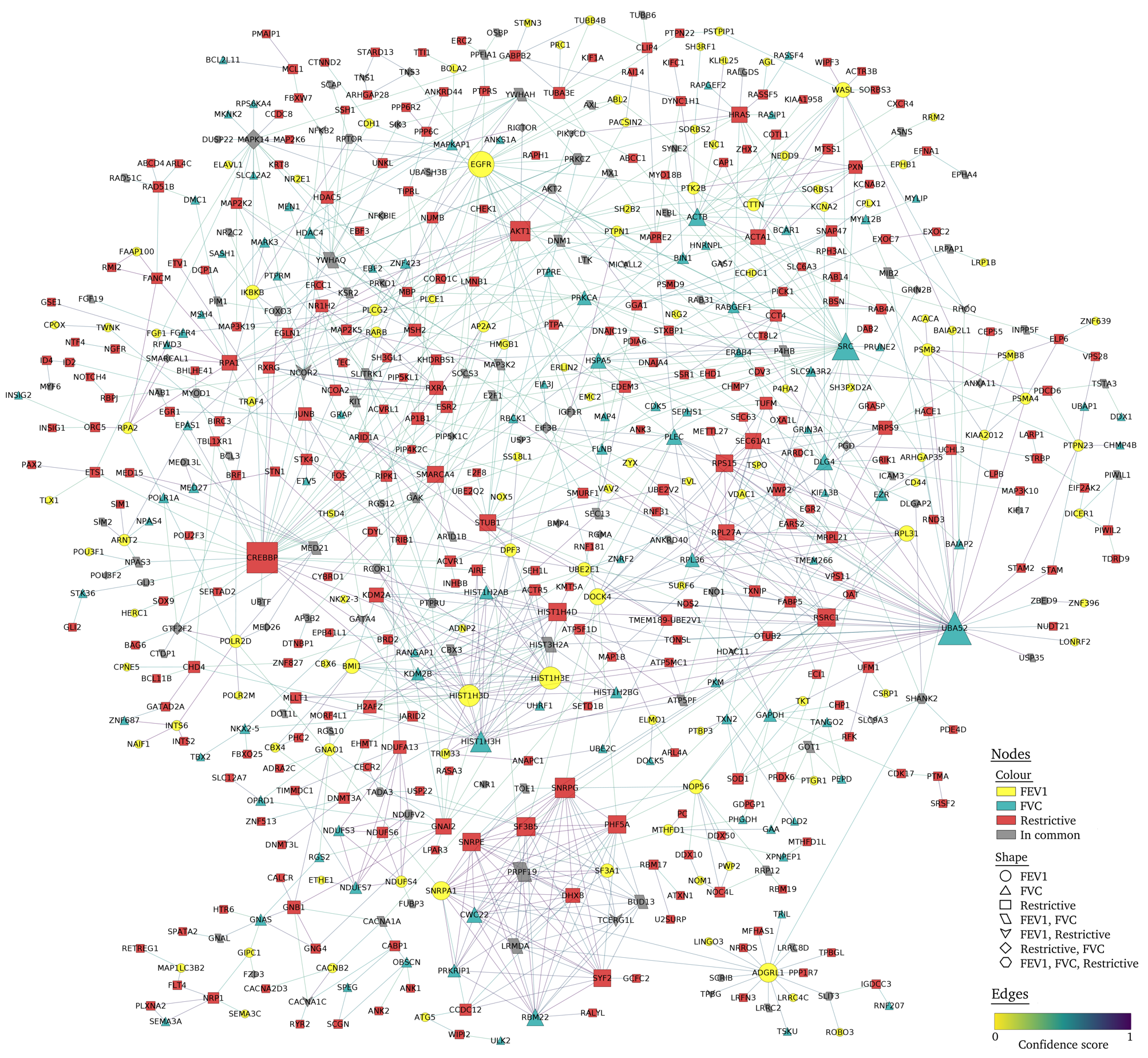

Supplement: Supplementary file 4 — Additional file 4. Figure S3. Protein-protein interaction networks for restrictive lung function phenotype: FEV1, FVC and restrictive vs normal lung function. [file 13148_2022_1294_MOESM4_ESM.pdf]
