# Supplementary material for: An epigenome-wide study of DNA methylation profiles and lung function among American Indians in the Strong Heart Study
Source: Clin Epigenetics. 2022 Jun 9;14:75. doi: 10.1186/s13148-022-01294-8 (PMC9185990; doi:10.1186/s13148-022-01294-8)
Supplement: Supplementary file 3 — Additional file 3. Figure S2. Protein-protein interaction network for airflow limitation phenotype: FEV1, FEV1/FVC and airflow limitation vs normal lung function. [file 13148_2022_1294_MOESM3_ESM.pdf]

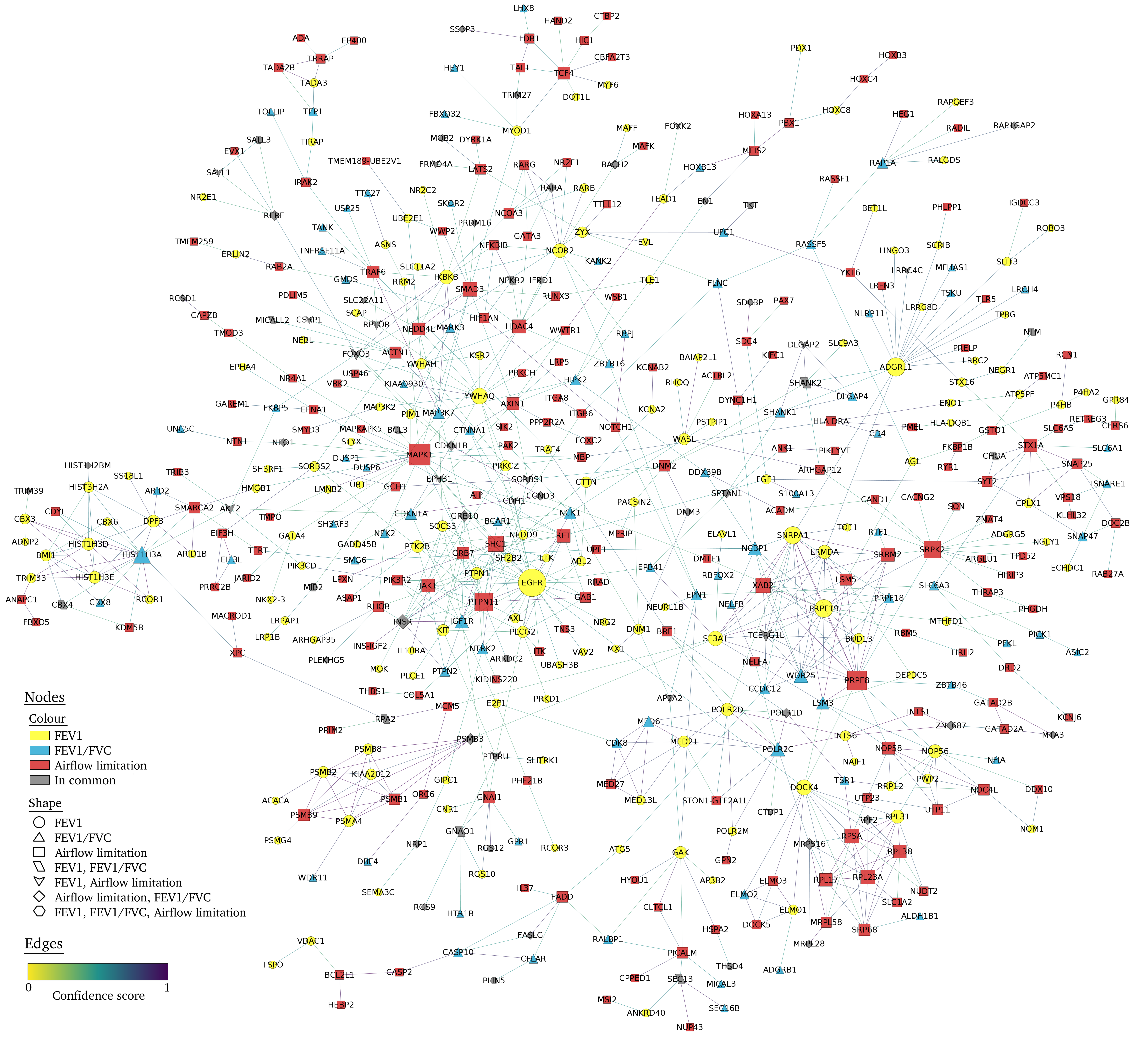
Nodes
Colour
FEV1
FEV1/FVC
Airflow limitation
In common
Shape
FEV1
FEV1/FVC
Airflow limitation
FEV1, FEV1/FVC
FEV1, Airflow limitation
Airflow limitation, FEV1/FVC
FEV1, FEV1/FVC, Airflow limitation
Edges
0
1
Confidence score
LHX8
HAND2
CTBP2
SSBP3
LDB1
HIC1
ADA
EP400
TRRAP
CBFA2T3
TADA2B
TAL1
TCF4
HEY1
MYF6
TADA3
TRIM27
DOT1L
TOLLIP
TEP1
FBXO32
MYOD1
SALL3
TIRAP
MOB2
DYRK1A
EVX1
SALL1
IRAK2
TMEM189-UBE2V1
FRMD4A
LATS2
RARG
NR2F1
BACH2
MAFF
MAFK
FOXK2
HOXA13
PBX1
MEIS2
HOXB13
PDX1
HOXB3
HOXC4
HOXC8
HEG1
RAPGEF3
RADIL
RAP1GAP2
RAP1A
RALGDS
RASSF1
NR2E1
RERE
TTC27
USP25
NR2C2
UBE2E1
SKOR2
RARA
RARB
PRDM16
NCOA3
TTLL12
TEAD1
EN1
TKT
BET1L
PHLPP1
IGDCC3
ROBO3
TANK
WWP2
GATA3
ZYX
EVL
UFC1
RASSF5
TMEM259
ERLIN2
TNFRSF11A
ASNS
NFKBIB
NCOR2
KANK2
LINGO3
SCRIB
SLIT3
RAB2A
GMDS
TRAF6
SLC11A2
RRM2
IKBKB
NFKB2
IFRD1
TLE1
FLNC
YKT6
LRRC4C
MFHAS1
RCSD1
PDLIM5
SMAD3
RUNX3
WSB1
LRFN3
TSKU
LRCH4
CAPZB
SLC22A11
SCAP
HIF1AN
HDAC4
SDCBP
PAX7
LRRC8D
TLR5
TPBG
TMOD3
MICALL2
CSRP1
RPTOR
NEDD4L
MARK3
WWTR1
RBPJ
NLRP11
NTM
NEBL
FOXO3
ACTN1
KSR2
SDC4
DLGAP2
SLC9A3
ADGRL1
PRELP
RCN1
LRRC2
EPHA4
USP46
YWHAH
LRP5
ZBTB16
KCNAB2
KIFC1
BAIAP2L1
ACTBL2
STX16
NEGR1
ATP5MC1
NR4A1
VRK2
PRKCH
HIPK2
RHOQ
SHANK2
DLGAP4
ATP5PF
P4HA2
GPR84
KIAA0930
YWHAQ
DYNC1H1
ENO1
P4HB
GAREM1
FKBP5
EFNA1
MAP3K2
AXIN1
ITGA8
KCNA2
SHANK1
HLA-DRA
HLA-DQB1
RETREG3
CERS6
PIM1
MAP3K7
ITGB6
PSTPIP1
GSTO1
SLC6A5
UNC5C
SMYD3
MAPKAPK5
BCL3
CTNNA1
SIK2
PPP2R2A
NOTCH1
WASL
CD4
PMEL
STX1A
NTN1
NEO1
STYX
CDKN1B
PAK2
FOXC2
ANK1
FKBP1B
SLC6A1
MAPK1
TRAF4
MBP
PIKFYVE
AGL
CHGA
DUSP1
DUSP6
PRKCZ
DNM2
ARHGAP12
RYR1
SNAP25
HIST1H2BM
SS18L1
TRIB3
SORBS2
SH3RF1
EPHB1
SORBS1
DDX39B
TSNARE1
TRIM39
HIST3H2A
ARID2
HMGB1
UBTF
LMNB2
GCH1
CTTN
FGF1
S100A13
SYT2
CBX3
CDYL
CBX6
DPF3
SMARCA2
AKT2
AIP
CDH1
CCND3
SPTAN1
CAND1
CACNG2
SON
CPLX1
VPS18
KLHL32
TMPO
CDKN1A
GRB10
NCK1
PACSIN2
DNM3
ACADM
ZMAT4
DOC2B
ADNP2
HIST1H3D
EIF3H
SH3RF3
NEK2
BCAR1
TOE1
RTF1
HIST1H3A
ARID1B
TERT
GATA4
SOCS3
SHC1
NEDD9
RET
MPRIP
ELAVL1
SNRPA1
LRMDA
SRRM2
SRPK2
ADGRG5
NGLY1
SNAP47
BMI1
EIF3L
SMG6
GADD45B
PTK2B
GRB7
UPF1
NCBP1
TPD52
ARGLU1
ECHDC1
TRIM33
HIST1H3E
JARID2
PIK3CD
SH2B2
LTK
DMTF1
HIRIP3
RAB27A
PRRC2B
LPXN
PTPN1
EGFR
ABL2
EPB41
RBFOX2
LSM5
SLC6A3
ANAPC1
RCOR1
NKX2-3
MIB2
ASAP1
PIK3R2
JAK1
RRAD
XAB2
THRAP3
CBX4
CBX8
LRPAP1
RHOB
PTPN11
GAB1
EPN1
NELFB
PRPF18
FBXO5
KDM5B
MACROD1
INSR
NEURL1B
PRPF19
PHGDH
LRP1B
ARHGAP35
IGF1R
AXL
NRG2
DNM1
SF3A1
TCERG1L
MTHFD1
KIT
PLCG2
TNS3
BRF1
BUD13
RBM5
PICK1
XPC
INS-IGF2
IL10RA
NTRK2
ITK
VAV2
MX1
NELFA
HRH2
PFKL
ASIC2
PLEKHG5
MOK
ARRDC2
UBASH3B
WDR25
PRPF8
DRD2
PLCE1
PTPN2
KIDINS220
CCDC12
DEPDC5
ZBTB46
THBS1
COL5A1
PRKD1
AP2A2
LSM3
GATAD2B
MCM5
E2F1
POLR2D
POLR1D
INTS1
KCNJ6
PRIM2
RPA2
MED6
ZNF687
GATAD2A
MTA3
PSMB3
CDK8
MED21
POLR2C
INTS6
PSMB8
PTPRU
SLITRK1
NOP58
NOP56
NAIF1
NFIA
PSMB2
KIAA2012
GIPC1
PHF21B
MED27
MED13L
STON1-GTF2A1L
DOCK4
TSR1
PWP2
RRP12
NOC4L
DDX10
PSMB1
ORC6
UTP23
UTP11
ACACA
PSMB9
PSMA4
CNR1
GNAI1
CTDP1
RPL31
NOM1
PSMG4
GNAO1
GPR1
POLR2M
RPF2
NRP1
RGS12
MRPS16
RPSA
RPL38
RCOR3
ATG5
GAK
GPN2
DBF4
RGS10
HYOU1
ELMO3
AP3B2
RPL17
RPL23A
NUDT2
WDR11
SEMA3C
IL37
FADD
ELMO2
ELMO1
SLC1A2
RGS9
HTR1B
CLTCL1
DOCK5
MRPL58
SRP68
ALDH1B1
HSPA2
MRPL28
VDAC1
FASLG
RALBP1
CASP10
CFLAR
PICALM
THSD4
ADGRB1
TSPO
BCL2L1
CASP2
PLIN5
CPPED1
SEC13
MICAL3
MSI2
ANKRD40
SEC16B
HEBP2
NUP43
